# Flavonoids from the Stems of *Croton caudatus* Geisel. var. *tomentosus* Hook

**DOI:** 10.3390/molecules15031097

**Published:** 2010-02-26

**Authors:** Guo-An Zou, Zhi-Heng Su, Hong-Wu Zhang, Yuan Wang, Jun-Shan Yang, Zhong-Mei Zou

**Affiliations:** Institute of Medicinal Plant Development, Chinese Academy of Medical Sciences and Peking Union Medical College, Beijing 100193, China; E-Mails: guoanzou@hotmail.com (G.-A.Z); hwzhang@implad.ac.cn (H.-W.Z); jsyang@implad.ac.cn (J.-S.Y.);

**Keywords:** *Croton caudatus* Geisel. var*. tomentosus* Hook, Euphorbiaceae, flavonoids, crotoncaudatin

## Abstract

A new flavone, named crotoncaudatin (**1**), was isolated from the stems of *Croton caudatus* Geisel. var. *tomentosus* Hook., together with nine known analogues: 3,5,6,7,8,3′,4′-heptamethoxyflavone (**2**), tangeretin (**3**), nobiletin (**4**), 5,6,7,4′-tetramethoxy-flavone (**5**), sinensetin (**6**), kaempferol (**7**), tiliroside (**8**), kaempferol-3-O-rutinoside (9) and rutin (**10**). The structures of the above compounds were established by a combination of spectroscopic methods, including HR-ESI-MS, ^1^H-NMR,^ 13C^-NMR, HMQC and HMBC spectra. All compounds were isolated from and identified in this species for the first time and compounds **1-6** are new for the genus *Croton*.

## 1. Introduction

The genus *Croton* belongs to the family of Euphorbiaceae, with more than 700 species in the World, widely distributed throughout tropical and subtropical regions. There are 21 species in China, mainly growing in the southern provinces [1]. It is well known as a source of diterpenoids because most of the species of this genus produce a significant variety of such terpenes (clerodane, labdane, kaurane, trachylobane, pimarane, *etc.*), regarded as the diagnostic ingredients with a wide range of biological activities such as anti-cancer [2], anti-inflammatory [3], and anti-ulcer [4]. However, only a few flavonoides have been reported from this genus [5,6,7]. 

*Croton caudatus* Geisel. var. *tomentosus* Hook. is a traditional Dai Nationalistic medicine, the stems and leaves of which have been used for the treatment of malaria, ardent fever, convulsions, rheumatic arthritis, and numbness [8]. It is one of the constituents in Qi Wei Ke Teng Zi Wan, which is a famous formula used by the Dai nationality of China for the treatment of pain and stomach diseases [9]. To date, however, no phytochemical investigation has been reported for *C. caudatus* Geisel. var. *tomentosus* Hook, except that done by our group [10]. Further investigations on the chemical constituents of this species have now led to the isolation of a new flavone **1**, along with nine known ones **2-10**. We report herein the isolation and structural elucidation of these compounds. 

## 2. Results and Discussion

The 75% EtOH extract of the stems of *C. caudatus* Geisel. var. *tomentosus* Hook. was sequentially extracted with petroleum ether, chloroform and *n*-BuOH. The *n*-BuOH-soluble portion of this extract was subjected to repeated chromatographic separations [HPD-100 macroporous resin column chromatography, silica gel (200-300 mesh) column chromatography, and Sephadex LH-20 column chromatography] to yield a new compound, named crotoncaudatin (**1**), along with nine known ones **2**-**10**. The known flavonoids were readily identified as 3,5,6,7,8,3′,4′-heptamethoxyflavone (**2**) [11,12], tangeretin (5,6,7,8,4′-pentamethoxyflavone, **3**) [11,12], nobiletin (5,6,7,8,3′,4′-hexamethoxyflavone, **4**) [11,12], 5,6,7,4′-tetramethoxyflavone (**5**) [11,13], sinensetin (5,6,7, 3′,4′-pentamethoxyflavone, **6**) [11], kaempferol (**7**) [14], tiliroside (**8**) [15], kaempferol-3-O-rutinoside (**9**), and rutin (**10**), by comparing their physical and spectroscopic data with those reported in the literature.

Crotoncaudatin (**1**) was obtained as yellow needles. The positive HR-ESI-MS showed a [M+Na]^+^ ion peak at *m/z* 453.1127 and a [M+H]^+^ ion peak at *m/z* 431.1308, corresponding to the molecular formula C_22_H_22_O_9_ (calc. 453.1156 for C_22_H_22_O_9_Na and 431.1337 for C_22_H_2__3_O_9_, respectively), possessing twelve degrees of unsaturation, which was further confirmed by the ^1^H- and ^13^C-NMR data (Table 1). Compound **1** was suggested to be a flavone based on the physico-chemical properties, chromatography performance and UV absorption maxima at 263 and 371 nm. The combination of ^1^H-, ^13^C-NMR, and HMQC spectral data of **1 **indicated the presence of six methoxyl groups [δ_H_ 3.85(3H, s), 3.88(3H, s), 3.91 (3H, s), 3.94 (3H, s), 4.08 (3H, s), and 4.08 (3H, s), with the corresponding δ_C_ 61.9, 61.6, 56.2, 56.2, 61.6, and 61.6, respectively.], one oxygenated -CH_2_ group [δ_H_ 5.14 (2H, s, H-5) with the correlated δ_C_ 68.0 (C-5)], two aromatic protons [δ_H_ 7.02 (1H, s, H-1), and 7.35 (1H, s, H-4) with the relevant δ_C_ 105.5 (C-1) and 109.2 (C-4), respectively], and thirteen quaternary carbons [δ_C_ 114.6 (C-7a), 118.1 (C-12b), 126.2 (C-4a), 136.8 (C-6a), 138.7 (C-11), 144.5 (C-9), 146.5 (C-11a), 146.9 (C-12a), 149.0 (C-8), 150.4 (C-2), 151.6 (C-10), 152.8 (C-3)] including a carbonyl carbon at δ_C_ 169.4 (C-7). The above mentioned data suggested compound **1** to be a hexamethoxylated flavone. In the HMBC spectrum, correlations were observed between δ_H_ 3.85 and δ_C_ 149.0 (C-8), δ_H_ 3.88 and δ_C_ 144.5 (C-9), δ_H_ 3.91 and δ_C_ 152.8 (C-3), δ_H_ 3.94 and δ_C_ 150.4 (C-2), δ_H_ 4.08 and δ_C_ 151.6(C-10), δ_H_ 4.08 and δ_C_ 138.7 (C-11), confirming the locations of the methoxyl groups. The long range correlations between δ_H_ 5.14 (H-5) and δ_C_ 105.2 (C-1), 118.1 (C-12b), 126.2 (C-4a), and 136.8 (C-6a) suggested a cyclization between the ring-B and ring-C through a –OCH_2 _group. Other key HMBC correlations are shown in Table 1. From the above described spectral evidence, compound **1** was identified conclusively as 2,3,8,9,10,11-hexamethoxy-7-oxo-[2]benzopyrano[4,3-b] [1] benzopyran. All the assignments of ^1^H- and ^13^C-NMR data for **1** were achieved by HMQC and HMBC experiments. Compound **1** represents a very rare group of flavonols with specific cyclization between 3-OH and C-2′ of ring B, so far reported from other plant families like Fabaceae or Caesalpiniaceae [16].

**Table 1:** ^1^H- (500 MHz), ^13^C-NMR (125 MHz) and HMBC data of **1** in (CD_3_)_2_CO.

Position	*δ_H_ J (Hz)*	*δ_C_*	HMBC
1	7.02 (1H, s)	105.5	C-4a,C-12a,C-3
2		150.4	
3		152.8	
4	7.35 (1H, s)	109.2	C-5,C-12b,C-2
5	5.14 (2H, s)	68.0	C-4,C-12b,C-4a,C-6a
7		169.4	
8		149.0	
9		144.5	
10		151.6	
11		138.7	
4a		126.2	
6a		136.8	
7a		114.6	
11a		146.5	
12a		146.9	
12b		118.1	
MeO-2	3.94 (3H, s)	56.2	C-2
MeO-3	3.91 (3H, s)	56.2	C-3
MeO-8	3.85 (3H, s)	61.9	C-8
MeO-9	3.88 (3H, s)	61.6	C-9
MeO-10	4.08 (3H, s)	61.6	C-10
MeO-11	4.08 (3H, s)	61.6	C-11

**Figure 1:**
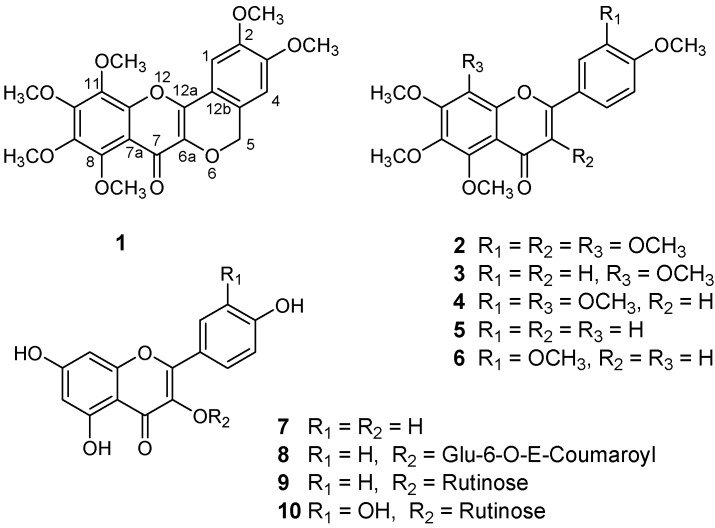
Structures of compounds **1**-**10**.

## 3. Experimental Section

### 3.1. General

Melting points were determined on a Fisher-Johns apparatus and were uncorrected. UV spectra were measured with Shimadzu UV-2550 UV-VIS spectrophotometer. IR spectra were recorded on Shimadzu FTIR-8400s. EI-MS spectrum was taken on Shimadzu GCMS QP2010 and HR-ESI-MS spectra were measured on LTQ Orbitrap XL spectrometer (Thermo Scientific). All NMR experiments were performed on a Bruker AM-500 spectrometer (Switzerland) (500 MHz for^ 1^H and 125 MHz for ^13^C). HPD-100 macroporous resin (Cangzhou Baoen Chemical Co., Ltd, China), Silica gel (Qingdao Haiyang Chemical Co., Ltd, China), Sephadex LH-20 (Amersham Pharmacia Biotech, Sweden) and preparative TLC (Yantai, China) were used for chromatography.

### 3.2. Plant material

The stems of *Croton caudatus* Geisel. var. *tomentosus* Hook. were collected from Xishuangbanna, Yunnan province, China, in August 2000 and identified by Prof. Zai-Lin Li, Yunnan Branch, Institute of Medicinal Plant Development (IMPLAD), Chinese Academy of Medical Sciences and Peking Union Medical College. A voucher specimen (YN2000B) is deposited at the Herbarium of IMPLAD.

### 3.3. Extraction and isolation

Dried powdered stems of *C. caudatus* Geisel. var. *tomentosus* Hook. (5 kg) were extracted with 95% EtOH (2 × 25 L) and then with 75% EtOH (2 × 25 L). After removal of the solvent under vacuum, 75% EtOH extract (277 g) was suspended in water (1 L) and partitioned successively with petroleum ether, chloroform and *n*-BuOH (each 3 × 1 L). The *n*-BuOH extract was dissolved in water and chromatographed over a HPD-100 macroporous resin column, eluted with H_2_O, 10%, 30%, 60%, and 95% EtOH in sequence to yield five fractions. The 95% EtOH eluate (4 g) was subjected to silica gel (100-200 mesh, 80 g) column chromatography, eluted with CHCl_3_-MeOH (from 19:1 to 0:1) in a gradient manner to yield 33 fractions, among which fractions 1~3 (0.5 g) were subjected to a silica gel (200-300 mesh, 15 g) column chromatography using petroleum ether-acetone (from 9:1 to 0:1) step-gradient elution to afford 62 fractions. Subfractions 8-17 were then subjected to Sephadex LH-20 column chromatography combined with preparative TLC (CHCl_3_-Me_2_CO = 10:1) to yield **2** (10 mg) and **3** (9 mg). Compounds **4** (10 mg) and **1** (4 mg) were obtained from the fractions 18~26 and 27~33 respectively by Sephadex LH-20 and preparative TLC (CHCl_3_-Me_2_CO = 10:1, and petroleum ether-Me_2_CO = 7:3, respectively). Compounds **5** (5 mg) and **6** (5 mg) were obtained from the fractions 34~39 and 50~53 by preparative TLC (petroleum ether-Me_2_CO = 3:2 and CHCl_3_-Me_2_CO = 6:1, respectively). The 60% EtOH eluate (9 g) was subjected to the silica gel (100–200 mesh, 400 g) column chromatography, eluted with CHCl_3_-MeOH (from 9:1 to 0:1) in a gradient manner to yield 32 fractions. Compounds **7** (4 mg), **8 **(15 mg), **9**(6 mg) and **10** (5 mg) were obtained from the fractions 4~9, 16~19, and 20~21 in the same way as mentioned above. The solvent system CHCl_3_-MeOH (1:1) was used as the eluent on Sephadex LH-20 CC in the whole experiment. 

Crotoncaudatin (**1**). Light yellow needle (petroleum ether-Me_2_CO), mp 182–184 °C，UV (MeOH) λ max: nm (log ε): 209 (4.6), 263 (4.3), 371 (4.1); IR (KBr) νmax cm^-1^: 2939 (C-H), 1628 (C=O), 1605 (C=C), 1464 (CH), 1059 (C-O) ; EI-MS *m/z* (rel int %): 430 (M^+^, 28), 429 (3), 416 (22), 415 (100), 402 (5), 401 (6), 399 (8), 387 (11), 372 (14), 371 (10), 357 (8), 341 (4), 197 (3); HR-ESI-MS (positive) *m/z*: 453.1127 [M+Na]^+^ and 431.1308 [M+H]^+^, (Calc. 453.1156 and 431.1337 for C_22_H_2__2_O_9_Na and C_22_H_2__3_O_9_, respectively). ^1^H- and ^13^C-NMR spectral data see Table 1. 

## 4. Conclusions

Previous reports have shown that the species of the genus *Croton* are rich in diverse diterpenoids, generally regarded as its characteristic chemical constituents. However, during our systematic chemical investigation on *C. caudatus* var.* tomentosus*, none of such components was isolated, instead, 10 flavonoids, especially polymethoxylated flavones (compounds **1**-**6**) were obtained, consistent with some reports that polymethoxylated flavones were also presented in *C. schiedeanus* [5], *C. ciliatoglanduliferus* [6], *C. brasiliensis* [7], and *C. cajucara* [17]. Moreover, this is not the first report of the absence of diterpenoids from the genus *Croton*, e.g. nearly none of the American *Croton* species has been reported to produce diterpenoids except *C. draco* [18]. Thus, the polymethoxylated flavones may possibly serve as useful chemotaxonomic markers for species of this genus. 
